# Superior Performance in Skilled Golfers Characterized by Dynamic Neuromotor Processes Related to Attentional Focus

**DOI:** 10.3389/fpsyg.2021.633228

**Published:** 2021-02-16

**Authors:** Kuo-Pin Wang, Cornelia Frank, Yen-yu Tsai, Kao-Hung Lin, Tai-Ting Chen, Ming-Yang Cheng, Chung-Ju Huang, Tsung-Min Hung, Thomas Schack

**Affiliations:** ^1^Center for Cognitive Interaction Technology (CITEC), Bielefeld University, Bielefeld, Germany; ^2^Neurocognition and Action – Biomechanics Research Group, Faculty of Psychology and Sports Science, Bielefeld University, Bielefeld, Germany; ^3^Sports and Movement Group, Department of Sports and Movement Sciences, School of Educational and Cultural Studies, University of Osnabrück, Osnabrück, Germany; ^4^Department of Physical Education, National Taiwan Normal University, Taipei, Taiwan; ^5^School of Psychology, Shanghai University of Sport, Shanghai, China; ^6^Graduate Institute of Sport Pedagogy, University of Taipei, Taipei, Taiwan; ^7^Institute for Research Excellence in Learning Science, National Taiwan Normal University, Taipei, Taiwan

**Keywords:** electroencephalography, precision sports, attention, constrained action hypothesis, meshed control theory

## Abstract

The meshed control theory assumes that cognitive control and automatic processes work together in the natural attention of experts for superior performance. However, the methods adopted by previous studies limit their capacity to provide in-depth information on the neuromotor processes. This experiment tested the theory with an alternative approach. Twelve skilled golfers were recruited to perform a putting task under three conditions: (1) normal condition, with no focus instruction (NC), (2) external focus of attention condition (EC), and (3) internal focus of attention condition (IC). Four blocks of 10 putts each were performed under each condition. The putting success rate and accuracy were measured and electroencephalographies (EEGs) were recorded. The behavioral results showed that the NC produced a higher putting success rate and accuracy than the EC and IC. The EEG data showed that the skilled golfers’ attentional processes in the NC initially resembled those in the EC and then moved toward those in the IC just before putting. This indicates a switch from more automatic processes to cognitive control processes while preparing to putt. The findings offer support for the meshed control theory and indicate the dynamic nature of neuromotor processes for the superior performance of athletes in challenging situations.

## Introduction

Attentional focus is a crucial factor in superior skilled performance (Wulf and Su, 2007; Wulf and Lewthwaite, 2016). In recent years, a number of research studies have specifically examined the internal vs. the external categories of attentional focus (Wulf and Prinz, 2001). An internal focus refers to attention being directed toward specific body actions, whereas an external focus relates to the effect that those body actions have on the environment (Wulf et al., 1998). A growing number of studies using attentional instructions have consistently demonstrated the advantage for motor performance of adopting an external focus relative to an internal focus (Bell and Hardy, 2009; Kearney, 2015). For instance, an external focus has been found to increase movement effectiveness, such as accuracy (Wulf and Su, 2007; Bell and Hardy, 2009), and movement efficiency, such as maximum force production (Marchant et al., 2011), speed, and endurance (Stoate and Wulf, 2011), in highly skilled performers. One possible explanation for the advantages of an external focus is provided by the constrained action hypothesis (Wulf et al., 2001; Mcnevin et al., 2003). This hypothesis assumes that individuals who direct their attention internally during skill execution experience conscious control of their movements (i.e., constraints on the motor system), which interferes with automatic control processes. In contrast, individuals who direct their attention to the effects of the movement experience less movement control. It promotes the use of automatic processes during motor preparation (Wulf et al., 2001). However, Schücker et al. (2014) and Vitali et al. (2019) who defined focusing on physical sensations as being internal sensations observed that internal sensation does not impair performance. They suggested that the internal sensations do not disrupt automated motor processes. Given that the definition of internal focus is different from the original definition of Wulf et al. (1998), it is possible to focus internally in more than one way which may lead to different results.

Furthermore, an external focus can be further distinguished as proximal or distal (Mcnevin et al., 2003). A proximal focus is close to the body (i.e., the club motion and the clubface when putting in golf) whereas a distal focus is further from the body (i.e., the desired trajectory of the ball). Previous studies that have adopted attentional instructions to examine proximal and distal focus in skilled performance have demonstrated that a distal focus benefits performance effectiveness relative to a proximal focus in golf chipping (Bell and Hardy, 2009) and putting (Kearney, 2015). These findings suggested that performers tend to move their attention toward higher levels of representation (i.e., the ball trajectory) and engage in less conscious monitoring of the lower-level features of the action (i.e., the clubface and its motion), resulting in adequate cognitive resources for motor preparation and a better coordinated action (Wulf and Su, 2007). However, these studies only adopted dichotomous focus manipulations and may not represent the actual type of attentional processes. According to the meshed control theory, the cognitive control and automatic processes of experts under natural conditions work in a synergistic manner for superior performance (Christensen et al., 2016). Furthermore, cognitive control processes focus on the higher-order strategic control (e.g., the ball trajectory) and situation control (e.g., adjusting the body action appropriately). Although both foci of attention are different from each other, both involve similar cognitive control processing since elite performers intend to pay their attention to self-monitoring processes (Bortoli et al., 2012; Wang et al., 2019). That is, both foci of attention are similar to the moment-to-moment awareness in mindfulness. For example, skilled performers need to be aware to both their goal (i.e., the higher-order strategic control) and to their action (situation control), without disrupting automated motor processes on implementation control (e.g., the clubface and its motion). In qualitative studies, Oliver et al. (2020) observed that skilled golfers switched their attentional focus between an external focus of attention and an internal focus of attention during motor preparation. Similarly, Bahmani et al. (2019) uncovered those athletes who switched both foci of attention in a difficult situation showed superior skill execution. Given that the nature of attentional focus is a complex and dynamic aspect of the superior skilled performance of athletes, it is important to investigate attention over time during skilled performance. However, the methods adopted in the aforementioned behavioral research are limited as they cannot provide in-depth information regarding the neuromotor processes over time. Electroencephalography (EEG) is an ideal method to detect dynamic processes in the superior skilled performance of athletes.

Electroencephalography provides a high temporal resolution of neural activity and therefore provides a window into understanding the dynamics of neuromotor processes in preparation for action. Specifically, the alpha 2 band (10–12 Hz) has been associated with an internal and an external focus during motor preparation (Radlo et al., 2002; Ellmers et al., 2016). For example, Radlo et al. (2002) examined the effects of an external and internal attentional focus on electrocortical activity and observed that novices who adopted an external focus relative to an internal focus had lower left hemisphere (T7 and O1) and right temporal (T8 and O2) alpha power in a dart throwing task. This suggested that novices who adopted an external focus had similar EEG patterns to skilled archers in their superior performance (Landers et al., 1991), which indicated more efficient neural processing. Furthermore, measures of cortico-cortical communication are critical to the determination of psychomotor efficiency during motor preparation (Hatfield, 2018). EEG coherence, the connection between different cortical areas, is an excellent measure of cortico-cortical communication (Deeny et al., 2003, 2009). Specifically, higher coherence indicates stronger cotico-cortical communication, whereas lower coherence implies the functional autonomy of cortex. Ellmers et al. (2016) utilized T7 (verbal-analytical) with Fz (motor-planning) and T8 (visuospatial) with Fz in assessing attentional focus in a golf putting postural task. Ellmers et al. (2016) observed that adopting an external focus relative to an internal focus of attention in novices resulted in decreased Fz-T7 alpha 2 coherence. Ellmers et al. (2016) suggested that this decreased Fz–T7 alpha 2 coherence reflected less use of verbal-analytical processes, and thus more automatic processes, whereas increased Fz–T7 alpha 2 coherence would be associated with more cognitive control processes. Although Fz–T7 alpha 2 coherence is valid for assessing the neuromotor processes underlying attentional focus, the aforementioned evidence did not reveal the dynamics of cortico-cortical communication underlying the nature of attentional focus in skilled performers. The simultaneous measurements of both processes in two time windows (one from −2 to −1 s and one from −1 s to task execution) and in a normal focus condition (i.e., no instruction) compared to an internal and an external focus condition (i.e., instruction) is needed to shed light on the dynamic nature of attentional processes in skilled performance.

To summarize, the present study examines the meshed control theory, which assumes that cognitive control and automatic processes work together in attentional processes underlying superior performance (Christensen et al., 2016). We adopted an approach to compare the well-known psychological states induced by different attentional focus manipulations (i.e., an external focus vs. an internal focus) with unmanipulated attentional processes, which have not been examined in previous studies. In addition, Bernier et al. (2011) and Oliver et al. (2020) observed that skilled golfers commonly and naturally – without any manipulation – adopted a distal external focus (i.e., visualizing the trajectory of ball) during motor preparation in a challenging condition. Along these lines, we assume that skilled golfers naturally adopt a distal external focus (i.e., visualizing the trajectory of the ball) during motor preparation. Therefore, not manipulating a distal external focus could enable a better understanding of the nature of attentional focus processes in skilled golfers. To examine the dynamics of neuromotor processes in skilled performers during motor preparation, we used two time windows for detecting EEG coherence, one from −2 to −1 s before putting and one from −1 s to execution of the putt. In addition, based on previous research showing that lower Fz–T7 alpha 2 coherence is associated with an external focus (i.e., more automatic processes) relative to an internal focus of attention (Ellmers et al., 2016), we expected skilled golfers adopting an external focus relative to an internal focus to show decreased Fz–T7 alpha 2 coherence (e.g., less verbal-analytical processes), indicating more automatic processes. Furthermore, most importantly, given the meshed control theory and Williams et al. (2015) assumes attention shifts from an external focus to an internal focus associated with superior motor performance in skilled performers in challenging conditions, we expected skilled performers in a no instruction condition (NC; exhibiting the actual attentional processes without manipulation) to have initial Fz–T7 alpha 2 coherence similar to that in the external focus condition (EC; more automatic processes), and then to switch to a coherence similar to that in an internal focus condition (IC; more cognitive control processes) for superior performance.

## Materials and Methods

### Participants

The number of participants was determined by means of power analysis software (G*Power 3.1). Consistent with previous EEG study in attentional instruction (Ellmers et al., 2016), we set the following input parameters for using a repeated measures ANOVA with alpha = 0.05, power = 0.80, effect size = 0.33~0.50 (corresponds to *η*_P_^2^ = 0.10~0.20), and actual power = 0.80. The resulting sample size specification as in SPSS recommendation was *N* = 8~16. Being aware of the potential for power analysis biases in the neuroscience field (Albers and Lakens, 2018) and large samples of elite athletes are typically hard to recruit for scientific studies, 12 skilled golfers (four females, eight males; mean age = 21.08 ± 4.64) with a mean golf experience of 9.66 years (*SD* = 3.57) were recruited, with a mean handicap of 3.25 (*SD* = 0.97). According to United States Golf Association (USGA) statistics, a handicap range of 2.0–5.9 reflects golf skill that is above 87.7% of female elite golfers and 98.27% of male elite golfers in the country (United States Golf Association, 2018). Thus, the skilled golfers could be defined as elite golfers at a high competitive level (Swann et al., 2015; Scharfen and Memmert, 2019). In addition, all of the recruited participants met the following selection criteria: (1) no history of neurological disease, (2) right-handed (Oldfield, 1971). All participants gave an informed written consent, and the study was approved by the Research Ethics Committee of National Taiwan Normal University. All methods were carried out in accordance with the relevant guidelines and regulations of Research Ethics Committee.

### Measures

#### Golf Putting Task

The golfers were set a putting task similar to that used by Wang et al. (2019). The putting task was executed in the laboratory on an artificial putting green (600 cm × 90 cm). Participants used their own golf putters to putt regular-size white golf balls (4.27 cm diameter) towards a standard-size hole (diameter = 10.8 cm). We instructed participants to putt from a distance that was chosen to set a difficult task, such that the average of five putting success rate was 40–60% in the warm-up phase. For example, the individual putting distance was designated 40–60% putting success rate. All participants putted 300 cm in the beginning distance. They performed five putts and the distance was adjusted relying on whether the average of five putting success rate was 40–60% or not. If the success rate was between 40 and 60%, the putting distance was set at 300 cm. If the success rate was above 60%, the putting distance would increase 30 cm and then they performed extra five putts to ensure the success rate reached 40–60%. On the contrary, if the success rate was below 40%, the putting distance would decrease 30 cm and then they performed extra five putts to ensure the success rate reached 40–60%. After the appropriate putting distance was decided, the participant performed 40 putts in each condition. Furthermore, the average distance that was related to 40–60% success is mean = 302 ± 24 cm. To avoid the learning effect during the task, the ball placed on different points of the circumference of the individual putting distance. For measuring EEG activity during motor preparation, the motor preparation period was defined as the time between placing the putter behind the ball and initiating the backswing (Lam et al., 2010), with the event-marker initiated *via* an infrared sensor that detected the movement of the backswing during each trial. Putting performance was judged by using a measuring tape to measure the distance between the ball and the hole; when a ball was holed, we registered the putt as having a distance of 0 cm.

#### Experimental Conditions

Based on previous research on attentional focus induced by giving explicit instructions to skilled golfers (Bell and Hardy, 2009; Toner and Moran, 2011), our experimental design was similar to those of Bell and Hardy (2009) and Toner and Moran (2011) who adopted three conditions – no instruction, an external focus, and an internal focus – for understanding the effects of attentional focus on performance effectiveness. The three conditions represented a putting task in which participants received instructions to:

1. Putt as they normally would in the NC;
2. Focus on the position of the clubface in the EC;
3. Focus on adjusting direction with hand movements and feeling sensation of hand movement in the IC.

#### Manipulation Check

To ensure that all participants had adopted the focus as instructed, we asked the participants to rate their experience on a five-point Likert scale, ranging from 0 (*not at all*) to 4 (*very much*; Bell and Hardy, 2009). After each putt, participants were asked to report the extent to which they focused on three types of foci:

1. Distal focus – to what extent were you focusing on the ball path?
2. Proximal focus – to what extent were you focusing on adjusting direction with the clubface?
3. Internal focus – to what extent were you focusing on adjusting direction with hand movements and feeling sensation of hand movement?

### EEG Recording

Consistent with Kao et al. (2013) and Wang et al. (2020), we used an electro cap (Quik-Caps, Neuroscan, Charlotte, NC, United States) to record the EEG activity and followed the International 10–20 EEG system (Jasper, 1958). The ground electrode was at the FPz site. An average-ear reference offline was taken from the left mastoids (A1) and right mastoids (A2) in 32 scalp locations. We also recorded vertical and horizontal electrooculograms (VEOG and HEOG) in bipolar configurations located superior and inferior to the right eye and on the left and right orbital canthi. All EEG data were recorded with a band-pass filter that was set at 1–100 Hz with the notch filter at 60 Hz. The impedance at each electrode site was below 5 kῼ. The data were obtained at a sampling rate of 1,000 Hz using Neuroscan 4.5 software and stored using Neuroscan NuAmps acquisition amplifiers (Neuroscan, Charlotte, NC, United States).

### Procedure

Before the testing day, we asked the participants to not consume any food or beverages containing alcohol or caffeine for 24 h. Before beginning the experiment, the participants were informed of their right to withdraw from the study at any time, and they provided their informed consent to participate. They were then fitted with a Lycra electrode cap, and asked to practice putting from a beginning distance of 300 cm between the hole and ball on the green to calculate their individual putting distances (Chen et al., 2019). We followed that of Chen et al. (2019) and Wang et al. (2020), who adopted an individual task difficulty. It can ensure the same level of difficulty for each performer to control confounding factors. The individual putting distance was determined 40–60% putting success rate (i.e., a challenging condition). Although participants performed an unequal number of putts during warm-up (Mean = 19 ± 10 putts), fatigue and learning effects are not probable because golfers were skilled athletes (Wang et al., 2020). The participants were then shown a list of the action components associated with a distal, a proximal, and an internal focus of attention when putting, and asked for confirmation that those action components were familiar to them. Furthermore, we adopted the same order of conditions for each participant (starting with the NC, then the EC, and finally the IC) because Perkins-Ceccato et al. (2003) who adopted a counterbalance design in attentional focus instructions observed attention order effect (external-internal vs. internal-external). Specifically, the external focus of attention instructions first resulted in lower variability in overall condition than internal instructions first in skilled golfers. They suggested that an external focus of attention instruction first may decrease a potential confounding factor for the results. Furthermore, the participants then performed 40 putts (in four blocks of 10 putts) with each of instructions (NC, EC, and IC) separately. Before each set of 10 trials, the experimenter reminded the participants to adhere to the instruction. After each putt, they were asked to respond to the manipulation check questions on the visual analogue scale. To increase the reliability of the manipulation checks, the participants were also asked to describe precisely what they were focusing on in each condition.

### Data Analysis

#### Behavioral Data

To measure the performance outcome, we calculated the putting success rate per condition as the number of balls holed out of 40 putts. That is, a certain number of balls into the hole divided by the total putts in each condition. For example, if participant putt 10 balls into the hole in first condition, 10 balls divide by 40 balls, so we get 25% putting success rate in first condition. In addition, we followed that of Moore et al. (2012) who adopted mean radial error (MRE) as accuracy data, defined as a subject’s average distance between ball after putt and hole in centimeters. Zero was recorded and calculated in MRE on trials where the putt was holed.

#### EEG Data

Following Semlitsch et al. (1986), an EOG correction was applied to the EEG data to eliminate the effects of blinking. Furthermore, we set a band-pass FIR filter from 1 to 30 Hz with 12 dB/oct for the EEGs and EOG channels. EEG data collected in the 2 s before the putt and containing amplitudes exceeding ± 100 μV were eliminated from subsequent analysis (Kao et al., 2014). Fast Fourier transforms with a Hanning window were used to transform all of the trials for coherence analysis and to maintain minimum spectral leakage. Coherence was defined as |*Cxy*(*f*)|^2^, where:

$$C_{\mathrm{xy}}\left(f\right)=\frac{\sum_{i}\left\{Xi\left(f\right)-X\left(f\right)\right\}{\left\{Yi\left(f\right)-Y\left(f\right)\right\}}^{*}}{\sqrt{\sum_{i}{\left|Xi\left(f\right)-X\left(f\right)\right|}^{2}\sum_{i}{\left|Yi\left(f\right)-Y\left(f\right)\right|}^{2}}}$$

and where *Xi*(*f*) and *Yi*(*f*) represent the Fourier transforms of the time series for electrode sites *X* and *Y*, respectively. Coherence was calculated in 1 Hz frequency bins and averaged across the appropriate frequencies to obtain the coherence values for the bandwidths. The electrode pairings of interest were Fz–T7 and Fz–T8 for 10–12 Hz (Ellmers et al., 2016). We applied a Fisher *z*-transformation to ensure an approximately normal distribution across subjects before conducting the statistical analysis.

### Statistical Analysis

SPSS 24.0 software was used for statistical analysis. First, we ran Friedman’s ANOVAs to analyze the self-reported manipulation check responses for each of the three conditions. Furthermore, we used Friedman’s ANOVAs by ranks to analyze differences in participants who reported attentional strategies for each condition. Second, we ran Friedman’s ANOVAs by ranks to analyze the putting success rate and ran a repeated-measures ANOVA over the three conditions for and MRE to evaluate the behavioral performance. Third, we ran a 3 (condition: NC, EC, and IC) × 2 (time: T1 = −2,000 ~ −1,000 ms, T2 = −1,000 ~ 0 ms) × 2 (coherence site: Fz–T7, Fz–T8) repeated-measures ANOVA on the 10–12 Hz EEG data to assess the dynamic changes in neuromotor processes. Fourth, to ensure that only the 10–12 Hz band was altered by attentional focus while the other frequency bands remained the same (i.e., to check for frequency specificity), we analyzed the flanking EEG frequency bands (Cheng et al., 2015). For this purpose, we ran a 3 (condition: NC, EC, and IC) × 2 (time: T1 = −2,000 ~ −1,000 ms, T2 = −1,000 ~ 0 ms) × 2 (coherence site: Fz-T7, Fz-T8) repeated-measures MANOVA on the 4–7 Hz and 16–20 Hz EEG data.

When the ANOVA detected significant effects, we performed *post hoc* calculations of the least significant difference (LSD) and false discovery rate (FDR), with the latter used to control for inflation of the Type I error value due to the multiple comparisons. The alpha level was set at 0.05 for all analyses before FDR (Genovese et al., 2002).

## Results

### Manipulation Check

**Figure 1:**
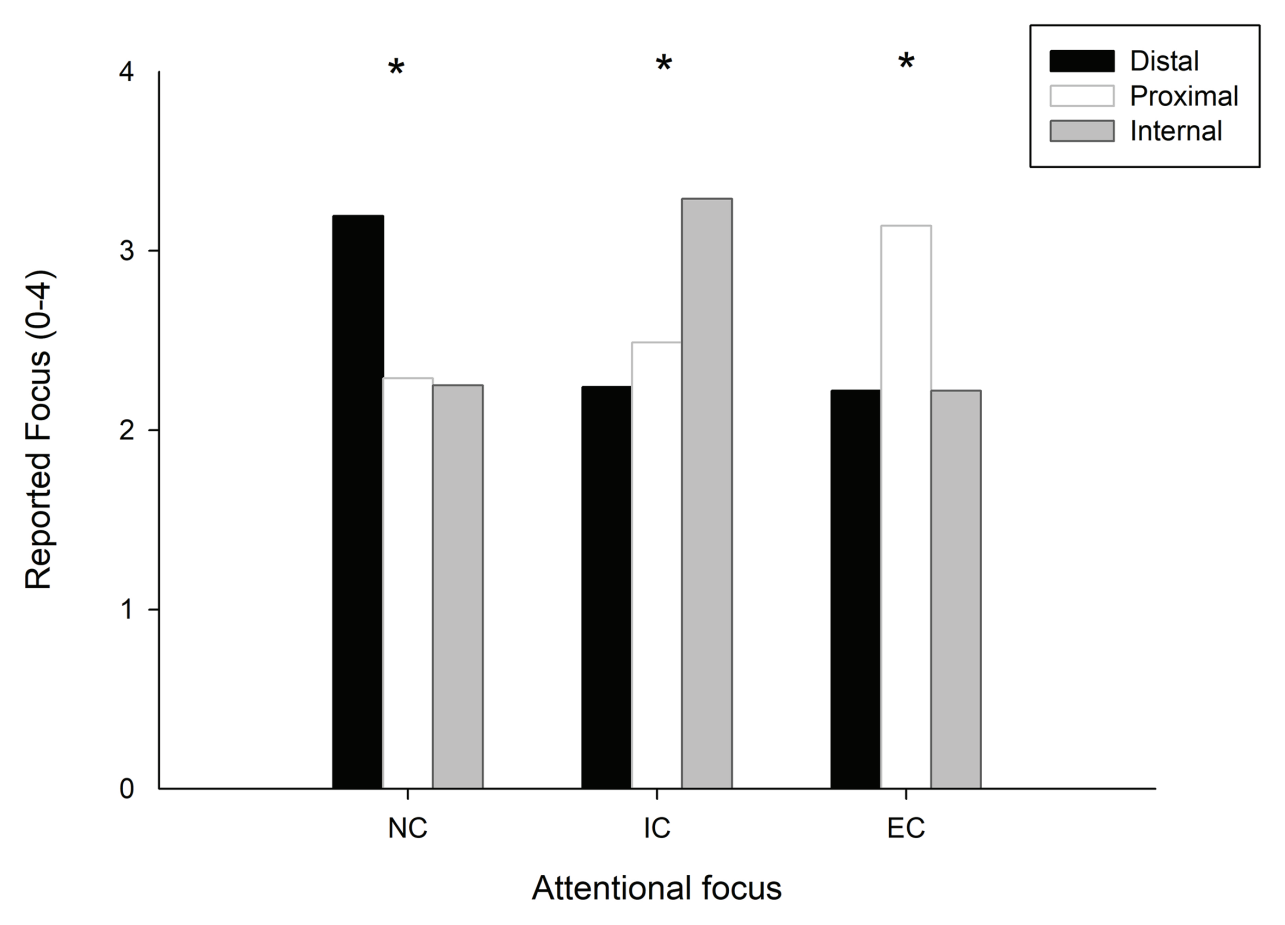
The self-reported manipulation check for each of the three attentional focuses. The participants rate their experience on a five-point Likert scale, ranging from 0 (not at all) to 4 (very much). NC, normal condition; IC, internal focus condition; and EC, external focus condition. *Signiﬁcant difference, p < 0.05.

There was a significant difference in the extent to which participants reported their attention on the three types of foci during motor preparation in no focus instruction condition, *ꭓ*^2^ (2, *N* = 12) = 12.809, *p* = 0.002, in internal focus of attention condition, *ꭓ*^2^ (2, *N* = 12) = 16.174, *p* < 0.001, and in external focus of attention condition (EC), *ꭓ*^2^ (2, *N* = 12) = 11.872, *p* = 0.003 separately. A follow-up Wilcoxon signed-rank test with FDR corrections revealed that the distal focus had a significantly higher rate than the internal focus (*p* = 0.005) and the proximal focus (*p* = 0.002) in the NC. No significant difference was observed between the internal focus and the proximal focus (*p* = 0.477). In the IC, the internal focus had a significantly higher rate than the distal focus (*p* = 0.003) and proximal focus (*p* = 0.002). Again, no significant difference was observed between the distal focus and proximal focus (*p* = 0.139). In the EC, the proximal focus had a significantly higher rate than the distal focus (*p* = 0.023) and internal focus (*p* = 0.002). There was no significant difference between the distal focus and internal focus (*p* = 0.756; Figure 1).

### Behavioral Results

#### Putting Success Rate and Accuracy

Friedman’s ANOVA by ranks revealed significant differences between the putting success rate (higher is better) in NC (Mdn = 2.67), EC (Mdn = 1.63), and IC (Mdn = 1.71), *ꭓ*^2^ (2, *N* = 12) = 9.415, *p* = 0.009. Follow-up pairwise comparisons conducted using Wilcoxon signed ranks tests revealed that the putting success rate in NC was significantly higher than that of EC (*p* = 0.024 FDR corrected) and IC (*p* = 0.043 FDR corrected). No statistically significant difference was observed between the EC and IC (*p* = 0.527 FDR corrected).

In addition, the accuracy data with a repeated-measures ANOVA indicated a significant effect of condition, *F*(2,22) = 5.562, *p* = 0.011, *η_p_*^2^ = 0.336. *Post hoc* analysis indicated that the NC (*M* = 11.22 ± 4.22 cm) had better accuracy than the EC (*M* = 16.32 ± 6.63 cm, *p* = 0.049 FDR corrected) and IC (*M* = 14.61 ± 5.14 cm, *p* = 0.036 FDR corrected). No statistically significant difference was observed between the EC and IC (*p* = 0.20).

### EEG Parameters

**Figure 2:**
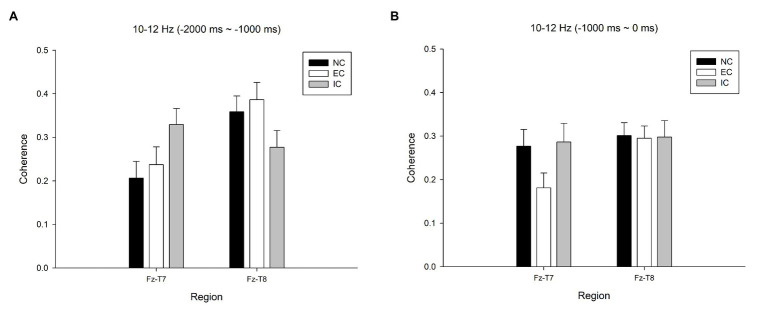
**(A)** Mean values (±SE) for 10–12 Hz coherence in the three conditions for the Fz-T7 and Fz-T8 electrode pairs in T1 (−2000 ~ −1,000 ms). **(B)** Mean values (±SE) for 10–12 Hz coherence in the three conditions for the Fz-T7 and Fz-T8 electrode pairs in T2 (−1,000 ~ 0 ms).

A 3 (condition: NC, EC, and IC) × 2 (time: T1 = −2,000~−1,000 ms, T2 = −1,000~0 ms) × 2 (coherence site: Fz–T7, Fz–T8) repeated-measures ANOVA for 10–12 Hz showed a significant Condition × Time × Coherence Site interaction, *F*(2,22) = 14.349, *p* < 0.001, *η_p_*^2^ = 0.566, Power = 0.996. A closer look at the simple effect analysis demonstrated a significant interactive effect in Condition × Coherence Site at T1, *F*(2,22) = 26.933, *p* < 0.001, *η_p_*^2^ = 0.710, and at T2, *F*(2,22) = 3.917, *p* = 0.034, *η_p_*^2^ = 0.265. A simple main effect analysis revealed a significant condition effect at Fz–T7 at T1, *F*(2,22) = 6.117, *p* = 0.008, *η_p_*^2^ = 0.357; Figure 2A, and at T2, *F*(2,22) = 5.417, *p* = 0.012, *η_p_*^2^ = 0.332; Figure 2B. Similarly, a simple main effect analysis revealed a significant condition effect at Fz–T8 at T1, *F*(2,22) = 5.316, *p* = 0.013, *η_p_*^2^ = 0.326. *Post hoc* analyses showed (a) higher Fz–T7 10–12 Hz coherence at T1 in the IC than in the NC (*p* = 0.024 FDR corrected) and EC (*p* = 0.043 FDR corrected); (b) lower Fz–T8 10–12 Hz coherence at T1 in the IC than in the EC (*p* = 0.036 FDR corrected) and NC (*p* = 0.048 FDR corrected); and (c) lower Fz-T7 10–12 Hz coherence at T2 in the EC than in the NC (*p* = 0.048 FDR corrected) and IC (*p* = 0.024 FDR corrected). As expected, skilled golfers in the NC showed dynamic changes in neuromotor processes.

### Control Analysis

#### Frequency Specificity

We compared adjacent frequency bands at Fz–T7 and Fz–T8 during motor preparation. The repeated-measures MANOVA on the 4–7 and 16–20 Hz data found no significant interaction effects: Condition × Time × Coherence site, Wilks’ lambda = 0.895, *F*(4,8) = 0.235, *p* = 0.911, *η_p_*^2^ = 0.105; Condition × Time, Wilks’ lambda = 0.567, *F*(4,8) = 1.528, *p* = 0.282, *η_p_*^2^ = 0.433; Condition × Coherence Site, Wilks’ lambda = 0.850, *F*(4,8) = 0.353, *p* = 0.835, *η_p_*^2^ = 0.150.

#### Learning and Fatigue Effect on EEG Coherence and Putting Performance

To eliminating the effects of learning and fatigue on brain activity and performance, we followed Ksao et al. (2013). Specifically, artifact-free trials and the putting accuracy data from each participant in each condition were divided into two parts (i.e., first 15 and last 15 putts) and averaged it. We hypothesized that the average Fz–T7 and Fz–T8 alpha 2, and putting accuracy were not significantly different between the first 15 putts and last 15 putts in each condition. First, we run a 2 (Putting Session: Session 1, Session 2) × (Coherence site: Fz–T7, Fz–T8) repeated-measures ANOVA on the 10–12 Hz EEG data in each condition separately. The results indicated no significant Putting Session × Coherence site interaction during the putting task in NC, *F*(1,11) = 0.103, *p* = 0.775, *η_p_*^2^ = 0.009, in EC, *F*(1,11) = 0.007, *p* = 0.936, *η_p_*^2^ = 0.001, and in IC, *F*(1,11) = 3.222, *p* = 0.100, *η_p_*^2^ = 0.227. Second, we run a paired *t* test in each condition to ensure that the putting performance did not change over time during the putting task in each condition. The results showed that the MRE was not significantly different between the first 15 putts and last 15 putts in the NC (*p* = 0.696), in EC (*p* = 0.286), and in IC (*p* = 0.657). Taken together, these results suggest that skilled golfers in present study did not change 10–12 Hz coherence and did not improve or decrease performance by accumulating trials. Thus, the control analysis eliminated the effects of learning and fatigue.

## Discussion

This study examined the dynamic neuromotor processes underlying the nature of attention in skilled golfers. We compared EEG coherence in three different focus conditions (i.e., no instruction condition; NC, external focus condition; EC, and internal focus condition; IC). The findings are consistent with those of previous work (Bernier et al., 2011; Fairbrother et al., 2016; Bahmani et al., 2019) and support the meshed control theory (Christensen et al., 2016). Our results showed that skilled golfers in the NC had similar Fz–T7 and Fz–T8 10–12 Hz coherence as they did in the EC, before switching to a state similar to the IC. Skilled golfers seem to operate with a self-regulated state of attention that optimally combines automatic and controlled processes. That is, superior performance cannot be directly improved by an external or internal focus instruction (Wulf, 2008). In addition, the results showed that adopting an internal focus of attention (i.e., being asked to focus on adjusting direction with hand movements and feeling sensation of hand movement) relative to an external focus of attention (i.e., being asked to focus on the position of the clubface) did not degrade performance, which suggests that skilled golfers use different types of information to stabilize their performance. The results also showed that adopting an external focus generated lower Fz–T7 10–12 Hz coherence, reflecting more automatic processes.

With regard to EEG coherence, lower Fz–T7 10–12 Hz coherence during motor preparation in EC compared to IC supports our hypothesis and corresponds with previous research. Ellmers et al. (2016) observed that novices who adopted an external focus relative to an internal focus had lower Fz–T7 10–12 Hz coherence in a postural task. That study suggested that performers who utilized an external focus could promote their automatic control processes because lower 10–12 Hz coherence at FZ–T7 has been associated with less verbal-analytic processes or language processing in motor planning (Deeny et al., 2003, 2009), which reflects more automatic processes (Cheng et al., 2017; Lo et al., 2019; Wang et al., 2020). However, our findings extend those of Ellmers et al. (2016) by showing that this holds not only for novices but also for skilled athletes. As such, our finding further specifies that external focus could promote their automatic control processes through 10–12 Hz coherence at Fz–T7 (i.e., verbal-analytic processes) in skilled athletes. Interestingly, we additionally found that skilled golfers in the EC had increased Fz–T8 10–12 Hz coherence at T1 (i.e., −2,000 ~ −1,000 ms) before putting. Increased Fz–T8 10–12 Hz coherence is associated with engaging in visuospatial processes in motor planning (Deeny et al., 2003, 2009). Although Fz–T8 10–12 Hz coherence may not be sensitive to changes in attentional focus in a simple voluntary sway task (Ellmers et al., 2016), it could detect changes in complex visuo-motor tasks (i.e., golf putting). Given that golf putting requires complex visuo-motor coordination, it is reasonable to suggest that Fz–T8 10–12 Hz coherence may be associated with visuospatial processing (i.e., a shift in focus on ball trajectory or end point) in motor planning.

Turning to the behavioral results between IC and EC, our findings contrasted with previous studies that have shown negative effects of internal focus in a golf chipping task (Wulf and Su, 2007; Bell and Hardy, 2009). Researchers have suggested that skilled performers’ movement control is relatively automatic and thus adopting an internal focus may engage unnecessary information processes, resulting in sub-optimal performance (Wulf et al., 2001). The constrained action hypothesis, an explanation for this phenomenon, assumes that individuals who direct their attention internally interfere with automatic control processes. This interfere constraints on movement, thus resulting in inferior performance (Wulf et al., 2001; Mcnevin et al., 2003). However, our finding is inconsistent with the conventional interpretation. This may be explained by two possible reasons. First, the definition of internal focus in present study is different from the original definition of Wulf et al. (1998). Wulf’s definition of internal focus of attention is that individuals direct their attention to control their actions in a relatively conscious movement. In contrast to Wulf’s definition of internal focus of attention, the definition of internal focus in present study is that individuals direct their attention to internal awareness on movement. In previous studies, Schücker et al. (2014) and Vitali et al. (2019) observed the attention focus on physical sensations does not disrupt performance. These findings suggested that performers who consciously monitor on their physical sensations did not constraint on the motor system, thus maintaining high-performance effectiveness under challenging conditions (Hanin and Hanina, 2009; Bortoli et al., 2012; Toner and Moran, 2015). Therefore, it is possibly leading to different results in our study. Second, the effects of attentional focus may be modulated by familiarity with attentional focus conditions in skilled performers (Maurer and Munzert, 2013). That is, highly practiced athletes may develop a specific skill-internal focus which does not have a disruptive influence on performance effectiveness (Toner and Moran, 2011). For instance, Maurer and Munzert (2013) observed that placing highly skilled performers in a familiar internal focus condition did not degrade performance effectiveness relative to a familiar external focus condition. Moreover, Wang et al. (2019) and Bertollo et al. (2016) revealed that skilled performers could allocate appropriate degrees of attention to the core components of action (i.e., adjustment of movements) for achieving optimal performance under a challenging task. Given that the present study ensured that skilled golfers were familiar with the internal focus instruction and the manipulation check also ensured that they adhered to internal focus instruction, we suggest that the use of adjusting direction with hand movements or feeling sensation of hand movement may not degrade performance effectiveness compared with adopting the proximal focus in skilled golfers.

In the NC, the data revealed that the skilled golfers most commonly adopted the strategy of focusing on the intended ball path as a distal external focus. Furthermore, the skilled performers operating under the NC showed similar results to EC processes at −2,000 ~ −1,000 ms, before switching to a similar state to that of IC processes just before executing the putt for superior performance. This finding extends previous qualitative studies, which have reported that athletes switched their attentional focus under challenging conditions for superior performance (Bernier et al., 2011; Fairbrother et al., 2016; Bahmani et al., 2019), and supports the meshed control theory. According to the meshed control theory, cognitive control and automatic processes work together to contribute to superior performance in challenging situations (Christensen et al., 2016). Cognitive control processes typically focus on the higher strategic control of the primary skill with its main goals (e.g., focusing on the ball trajectory and the hole when putting) and on the situational control with the control of action in immediate the situation (e.g., adjusting movement in the performance context). Meanwhile, automatic processes typically focus on implementation control that involves performing relatively stable actions (e.g., keeping clubface in the right direction). As such, taking a ball path focus involves visualizing a line from the ball with clubface (similar to an external focus) to the target, and then checking the final position (i.e., making technical adjustments or feeling sensation of the core action component) before putting (Wulf and Su, 2007; Kearney, 2015). There is no surprise in our finding that skilled golfers in the NC had dynamic neuromotor processes (Williams et al., 2015; Oliver et al., 2020). The finding not only supports the meshed control theory but also further specifies the dynamic neuromotor processes underlying the nature of attention in skilled golfers for superior performance.

In terms of implications for coaches and athletes, our findings suggest that no focus instruction in skilled performers may result in a superior cognitive-motor processing and performance when they face challenging situations (Beilock and Gray, 2007; Toner and Moran, 2015). In addition, our EEG results showed that the skilled golfers’ attentional processes initially resembled in an external focus of attention and then moved toward an internal focus of attention. We recommend that practitioners should encourage athletes to develop the attentional strategies including the familiarity with an external focus of attention and an internal sensation focus of attention during motor preparation in challenging situations (Bortoli et al., 2012; Bertollo et al., 2013).

Our control analysis showed frequency specificity at Fz–T7 and Fz–T8, and the manipulation check indicated that participants adhered to the instructions. Nonetheless, some limitations should be noted. First, our sample was relatively small compared with previous studies (e.g., Ellmers et al., 2016, *N* = 24; Radlo et al., 2002, *N* = 20) and thus, although our study was sufficiently powered to detect the interaction effects detailed above, the results should be interpreted with caution until they are replicated in a larger sample. Second, to improve the spatial resolution of the EEG, a high-density EEG recording and a source localization algorithm could be used in future studies to confirm the origin of Fz, T7, and T8. Third, to test the meshed control theory more thoroughly, future research should compare tasks at a range of difficulty levels (from easy to highly challenging) and include participants across a range of skill levels (from novices to skilled performers). This approach would provide a clearer overall picture of whether cognitive control processes come to play an increasing role in more highly skilled performers. Fourth, we acknowledge that not manipulating a distal external focus in our instruction may be a limitation in our research because a distal focus of attention is not only about visualizing the trajectory of the ball, but also towards a target (i.e., golf hole). It would be worth studying the differential effect of a distal external focus instruction and the actual type of attentional processes with EEG. It would provide more a comprehensive picture of the underlying mechanisms. Fifth, we adopted the same order of conditions in our study because Perkins-Ceccato et al. (2003) who adopted a counterbalance design observed that the external focus of attention instructions first resulted in lower variability in overall condition than internal instructions first in skilled golfers. This finding raises the concern that the external focus of attention instructions first may decrease a potential confounding factor for the results. However, in another study, Wulf and Su (2007) adopted the counterbalanced design to reduce order effects. These methodological differences may impact on the results. It is recommended that future studies should replicate Perkins-Ceccato et al. (2003) study to examine whether the different order of attentional instruction affects the performance in other precision sport (e.g., golf putting, dart throwing, and archery).

In conclusion, the present study extends previous findings by specifying that skilled performers receiving an external focus of attention instruction had reduced verbal-analytic processes (i.e., more neuromotor supported automatic processes) relative to when they received an internal focus of attention instruction. In addition, adopting an internal focus did not always degrade the performance of skilled performers executing a challenging task relative to adopting an external focus. This indicates that the action-related content of the focus plays a major role. Finally, the present study found that skilled performers receiving no focus instructions first adopted a state similar to external focus processes, which include reduced verbal-analytic and increased visuospatial processes, and then shifted to a state similar to internal focus processes, which include increased verbal-analytic processes, just before putting for superior performance. These findings not only support the meshed control theory but also highlight the neuro-temporal dynamics of these processes.

## Data Availability Statement

The original contributions presented in the study are included in the article/supplementary material, further inquiries can be directed to the corresponding authors.

## Ethics Statement

The studies involving human participants were reviewed and approved by The Research Ethics Committee of National Taiwan Normal University. Written informed consent to participate in this study was provided by the participants’ legal guardian/next of kin.

## Author Contributions

All authors listed have made a substantial, direct and intellectual contribution to the work, and approved it for publication.

### Conflict of Interest

The authors declare that the research was conducted in the absence of any commercial or financial relationships that could be construed as a potential conflict of interest.
